# Rare Sequelae of Endoscopic Retrograde Cholangiopancreatography: Polymicrobial Bloodstream Infection and Hepatic Abscess in an Elderly Individual

**DOI:** 10.7759/cureus.40517

**Published:** 2023-06-16

**Authors:** Hamad Ahmad, Urooj Khan, Hoore Jannat, Noaman Ahmad

**Affiliations:** 1 Internal Medicine, Westchester Medical Center, Valhalla, USA; 2 Internal Medicine, Khyber Medical University, Peshawar, PAK; 3 Internal Medicine, Khyber Medical College, Peshawar, PAK; 4 Internal Medicine, Huntsville Hospital, Huntsville, USA

**Keywords:** complications, polymicrobial bloodstream infection, hepatic abscess, ercp, endoscopic retrograde cholangiopancreatography

## Abstract

Endoscopic retrograde cholangiopancreatography (ERCP) is an advanced technique using a side-viewing upper endoscope to diagnose and treat pancreaticobiliary diseases. ERCP is generally considered a safe procedure; however, it is associated with risks of certain complications such as pancreatitis, bowel perforation, bleeding, and infections. Very rarely, ERCP can result in abscess formation in different organs, such as the pancreas, liver, and intestines. Physicians should be vigilant for rare post-ERCP complications such as clinically significant bacteremia and hepatic abscess, especially in high-risk populations, as if left untreated, they can result in significant morbidity and mortality. We present an interesting and rare case of an 80-year-old patient who presented with nausea, vomiting, and abdominal pain post-ERCP and was found to have a polymicrobial bloodstream infection and a hepatic abscess. The patient was treated with medical therapy alone, with an appropriate clinical response.

## Introduction

Endoscopic retrograde cholangiopancreatography (ERCP) is an advanced endoscopic procedure used for diagnosing and managing various pancreaticobiliary disorders [1]. During ERCP, a specialized side-viewing upper endoscope is guided into the duodenum to access the biliary and pancreatic ducts. The procedure involves injecting a contrast medium to visualize the ducts and perform therapeutic interventions. However, ERCP carries a higher risk of serious complications compared to other endoscopic procedures [2]. Reported rates of ERCP-related complications range from 7% to 12%, with mortality rates ranging from 0.1 to 1.4% [3]. Risk factors for complications include the difficulty of cannulation, sphincterotomy, percutaneous sphincterotomy, surgically altered anatomy, sphincter of Oddi dysfunction, periampullary diverticulum, cirrhosis, older age, and end-stage kidney disease [1]. Higher hospital and endoscopist procedure volumes have been associated with lower complication rates [4]. The most common serious complications of ERCP are pancreatitis, bleeding, perforation, and infections [5].

Infections following ERCP, if not diagnosed and treated promptly, can lead to severe complications, such as ascending cholangitis, which carries significant morbidity and mortality [3]. One of the potential complications arising from biliary tree infection is the development of a hepatic abscess, with a mortality rate as high as 12% if not treated promptly and appropriately [6]. Despite advances in technology and safety measures, procedure-related mortality rates have not decreased over time [5]. Early recognition of complications is crucial for prompt treatment. We report a rare case of an 80-year-old patient who initially presented with symptoms of nausea, vomiting, and abdominal pain, subsequently leading to the discovery of a polymicrobial bloodstream infection and the formation of a hepatic abscess after undergoing an ERCP procedure.

## Case presentation

**Figure 1:**
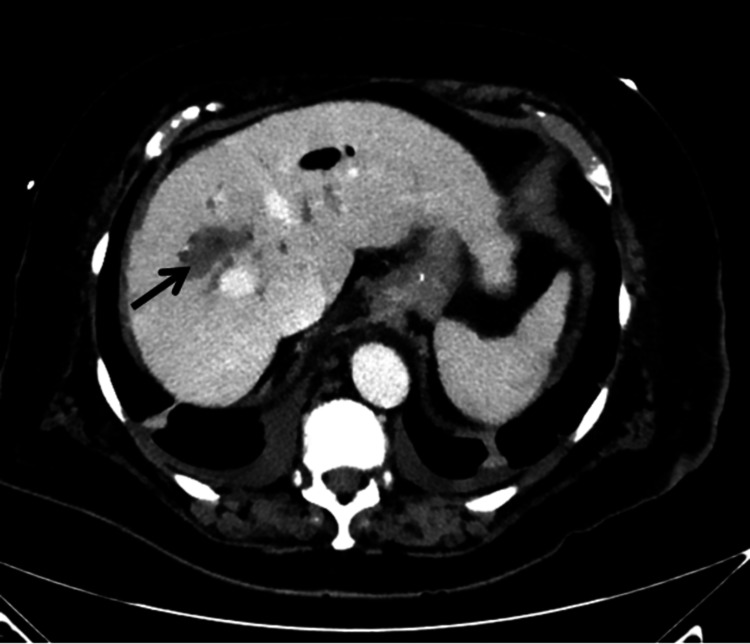
Computed tomography scan abdomen: poorly defined 3.4 cm × 2.3 cm hypoattenuating region with no peripheral enhancing lesion in the right hepatic lobe, indicating a hepatic abscess

**Figure 2:**
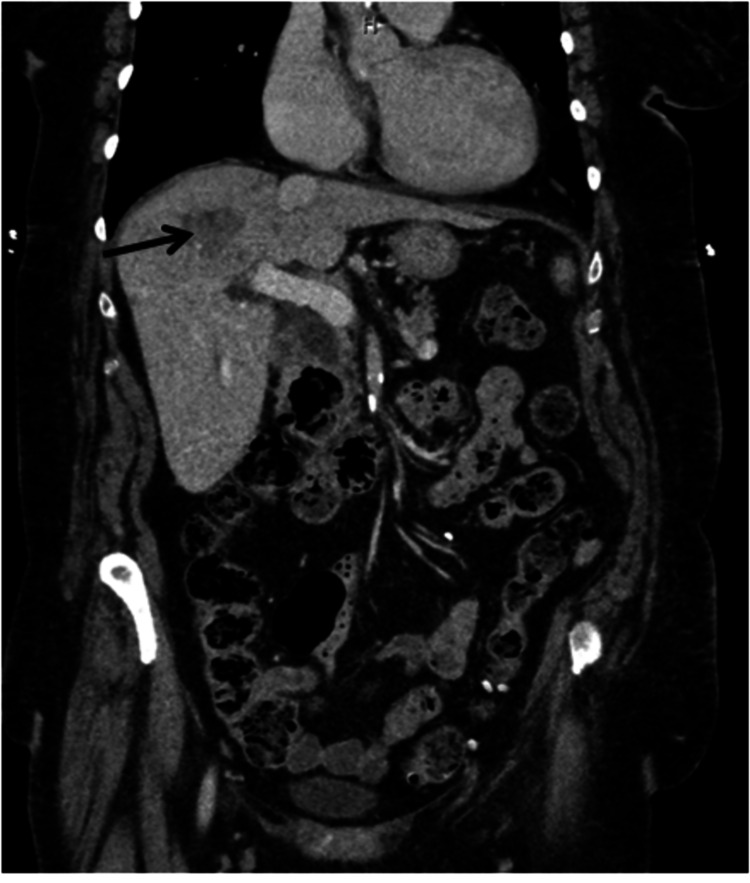
Computed tomography scan abdomen: poorly defined 3.4 cm × 2.3 cm hypoattenuating region with no peripheral enhancing lesion in the right hepatic lobe, indicating a hepatic abscess

An 80-year-old female patient presented with a significant medical history, including persistent atrial fibrillation, chronic obstructive pulmonary disease (COPD) not on steroids, hypertension, and rheumatoid arthritis not on immunosuppressants, with a recent hospitalization for choledocholithiasis requiring ERCP and biliary stent placement. The patient returned six weeks later for elective ERCP with stent removal and was discharged home. Five days later, the patient returned to the hospital with nausea, vomiting, and abdominal pain. On examination, the patient was not in acute distress, though she had mild tenderness over the right upper quadrant of the abdomen. Blood work revealed an elevated white blood cell count of 24 k/mm3 (normal: 4-11 k/mm^3^), mildly elevated aspartate aminotransferase 44 U/L (normal: 4-35 U/L), and alanine aminotransferase 66 U/L (normal: 5-60 U/L), and normal amylase, lipase, lactic acid, alkaline phosphatase, and total bilirubin. Blood cultures demonstrated polymicrobial bloodstream infections: Klebsiella pneumoniae and Enterococcus faecalis, both susceptible to multiple first-line antibiotics. The patient was started on ampicillin-sulbactam and monitored closely. A computed tomography (CT) scan showed a poorly defined 3.4 cm hypoattenuation region in the right hepatic lobe, suggestive of a hepatic abscess (Figures 1-2).

Given the multi-organism bacteremia, a transthoracic echocardiogram was performed, which revealed no signs of endocarditis. The patient responded appropriately to antibiotics, with a resolution of nausea and abdominal pain and a normalization of white cell count. A gastroenterologist assessed the patient and, given the improvement in symptoms and no signs of biliary obstruction based on normal total bilirubin and alkaline phosphatase levels, recommended no endoscopic interventions. Interventional radiology also did not attempt percutaneous drainage as the hypodense lesion was not well defined and encapsulated, and intervention would not necessarily be beneficial. Hence, the patient was treated conservatively with antibiotics only. Repeated blood cultures did not show any growth. Given the excellent clinical response, the patient was deemed medically stable for discharge home on amoxicillin-clavulanate for a total of a two-week antibiotic course with outpatient imaging follow-up. A repeat CT scan a month later demonstrated complete resolution of the abscess.

## Discussion

ERCP is widely used these days to diagnose and manage diseases related to the pancreaticobiliary system. The most common indication for ERCP is choledocholithiasis, as was the case in our patient. Other indications include acute cholangitis, drainage of malignant biliary obstruction, post-surgical biliary complications such as a biliary leak or stricture, management of acute pancreatitis-related complications such as pancreatic duct strictures or stones, and diagnosis and treatment of extrabiliary pathologies such as tissue biopsies for diagnostic purposes and therapeutic pseudocyst drainage [5]. Our patient had two ERCP procedures done: one for choledocholithiasis and biliary stent placement, and another for the biliary stent removal four weeks later. As with any endoscopic procedure, ERCP is associated with risks of complications; hence, consent should cover pertinent and significant adverse events associated with each specific ERCP procedure. Informed consent for ERCP should address at least these six possible complications: (a) acute pancreatitis, (b) bleeding, (c) infections, (d) cardiorespiratory events, (e) hypersensitivity reaction, and (f) perforation [1]. Clinically significant bacteremia post-ERCP occurs in up to 5% of patients [7-8]. Our patient had multiorganism bacteremia: Klebsiella pneumoniae and Enterococcus faecalis. ERCP duration longer than 60 minutes, tandem endoscopic ultrasound-endoscopic retrograde cholangiopancreatography with fine needle aspiration, and age above 75 years are significant risk factors for post-ERCP bacteremia [9]. Our patient, given her age, two ERCP procedures in a four-week period, and history of COPD and rheumatoid arthritis, was at high risk for infections, including bacteremia. Hepatic abscess is another rare complication after endoscopic procedures, with an increasing incidence [6]. In the study by Tsai et al. on 2135 patients with a primary diagnosis of hepatic abscess and 10,675 patients as reference controls without hepatic abscess, they found a higher rate of liver abscesses in patients with a history of recent upper gastrointestinal endoscopy [9]. The etiology of hepatic abscesses can be bacterial, parasitic, fungal, or mixed (pyogenic superinfection of a parasitic or fungal abscess) [10]. Our patient's hepatic abscess etiology was likely bacterial given concomitant bacteremia, an appropriate response to antibiotics, and no risk factors for parasitic or fungal infections. Similar to our patient, several cases of hepatic and para-hepatic abscesses have been reported after endoscopic procedures. Rafiq et al. reported a patient who developed multiple liver abscesses with liver necrosis after percutaneous endoscopic gastrostomy tube placement [11]. Unfortunately, due to the patient's worsening critical condition, the patient was managed only per comfort-care measures and did not undergo any interventions. Similarly, Ji et al. reported an interesting case when a patient developed a ligamentum teres hepatic abscess after an ERCP and underwent laparoscopic drainage of the abscess with complete recovery [7]. Sonography and/or CT scans are primarily used to diagnose hepatic abscesses, with needle aspiration for bacteriology studies to confirm the diagnosis if needed. [12]. Given the presence of bacteremia, the appropriate response to antibiotics, and the patient's age, we decided against needle aspiration. A history of COPD and rheumatoid arthritis made her a high-risk patient, and hence, we kept the threshold for additional procedures very high. The therapeutic strategy consists of bactericidal antibiotics, sometimes in combination with percutaneous or surgical drainage, and control of the primary source. Hope et al. reported a 100% complete resolution rate of hepatic abscesses less than 3 cm with antibiotic therapy alone in their series of 107 patients [13]. Similarly, in a literature review of 465 abscesses treated with medical therapy alone, 176 of which were located in the liver, they found that the 5 cm cut-off was the main factor associated with medical therapy success, and the success rate was over 80% for hepatic abscess resolution in this review [14]. Similar to the findings of Hope et al., our patient responded appropriately to medical therapy alone. Early criteria for effective treatment are apyrexia, the disappearance of pain, and normalization of leukocytosis and CRP; reversal of imaging findings usually occurs somewhat later [15].

## Conclusions

ERCP is a valuable diagnostic and therapeutic procedure for pancreaticobiliary diseases; however, like any endoscopic procedure, ERCP carries the risk of complications. In addition to the most common complications, physicians should be vigilant for rare complications such as clinically significant bacteremia and hepatic abscess, especially in high-risk populations (75 years, tandem EUS-ERCP with FNA, procedure duration >60 minutes). The incidence of bacteremia and hepatic abscesses post-endoscopic procedure is on the rise, and continued research and vigilance are necessary to further understand and prevent these rare complications and ensure optimal patient outcomes.
